# Recombinant Full-Length TDP-43 Oligomers Retain Their Ability to Bind RNAs, Are Not Toxic, and Do Not Seed TDP-43 Aggregation in Vitro

**DOI:** 10.1021/acschemneuro.3c00691

**Published:** 2023-12-20

**Authors:** Lixin Yang, Yllza Jasiqi, Hilal Lashuel

**Affiliations:** Laboratory of Molecular and Chemical Biology of Neurodegeneration, Institute of Bioengineering, Ecole Polytechnique Fédérale de Lausanne, 1015 Lausanne, Switzerland

**Keywords:** Neurodegenerative diseases, TDP-43 oligomers, aggregation, seeding, cytotoxicity

## Abstract

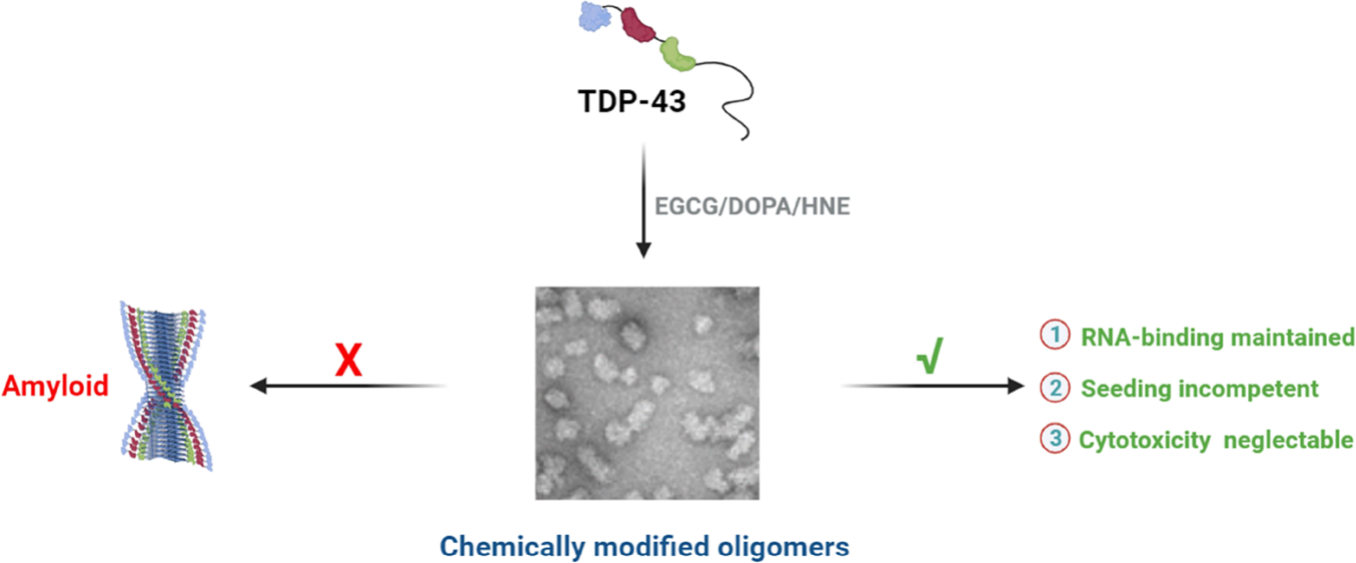

TAR DNA-binding protein with 43 kD (TDP-43) is a partially disordered protein that misfolds and accumulates in the brains of patients affected by several neurodegenerative diseases. TDP-43 oligomers have been reported to form due to aberrant misfolding or self-assembly of TDP-43 monomers. However, very little is known about the molecular and structural basis of TDP-43 oligomerization and the toxic properties of TDP-43 oligomers due to several reasons, including the lack of conditions available for isolating native TDP-43 oligomers or producing pure TDP-43 oligomers in sufficient quantities for biophysical, cellular, and in vivo studies. To address these challenges, we developed new protocols to generate different stable forms of unmodified and small-molecule-induced TDP-43 oligomers. Our results showed that co-incubation of TDP-43 with small molecules, such as epigallocatechin gallate (EGCG), dopamine, and 4-hydroxynonenal (4-HNE), increased the production yield of TDP-43 stable oligomers, which could be purified by size-exclusion chromatography. Interestingly, despite significant differences in the morphology and size distribution of the TDP-43 oligomer preparations revealed by transmission electron microscopy (TEM) and dynamic light scattering (DLS), they all retained the ability to bind to nucleotide DNA. Besides, circular dichroism (CD) analysis of these oligomers did not show much difference in the secondary structure composition. Surprisingly, none of these oligomer preparations could seed the aggregation of TDP-43 core peptide 279–360. Finally, we showed that all four types of TDP-43 oligomers exert very mild cytotoxicity to primary neurons. Collectively, our results suggest that functional TDP-43 oligomers can be selectively stabilized by small-molecule compounds. This strategy may offer a new approach to halt TDP-43 aggregation in various proteinopathies.

## Introduction

It has been estimated that in over 97% of patients with amyotrophic lateral sclerosis (ALS) and 45% of patients with frontotemporal lobar degeneration (FTLD), neuropathology is characterized by the presence of cytoplasmic TAR DNA-binding protein 43 (TDP-43) inclusions in brains, suggesting that TDP-43 plays an important role in the pathogenesis of these diseases.^1^ TDP-43 is a transcription factor and is usually found in aggregated and post-translationally modified forms (e.g., hyperphosphorylation, ubiquitination, and cleaved fragments).^2^ TDP-43 aggregates and inclusions also co-occur in many other neurodegenerative diseases (NDDs), including Alzheimer’s disease, Parkinson’s disease, Huntington’s disease, and the newly defined limbic-predominant age-related TDP-43 encephalopathy,^3^ which are collectively referred to as TDP-43 proteinopathies.^4^ Therefore, a better understanding of the mechanisms of TDP-43 aggregation, the nature of TDP-43 toxic species, and the molecular/cellular determinants of their formation has wide-ranging implications for the diagnosis and treatment of several NDDs.

TDP-43 (414 amino acids) is a partially disordered protein with a ubiquitin-like N-terminal domain (NTD), two folded RNA recognition motifs (RRMs), and a low-complexity C-terminal domain (CTD).^5^ The native state of TDP-43 is not fully understood, but it is accepted that native TDP-43 can form homodimers and exists in a monomer-dimer-oligomer equilibrium directed by the head-to-tail self-assembly of its NTDs.^6−9^ A study by Afroz et al. demonstrated that functional and dynamic oligomerization through the NTD of TDP-43 may antagonize its pathologic aggregation.^10^ Besides, several lines of evidence suggest that TDP-43 dimerization/oligomerization is necessary for its normal liquid–liquid phase separation and RNA splicing functions.^11−13^ TDP-43 oligomerization driven by RRMs or the C-terminal amyloid core region was also reported to be triggered by pathological mutations, post-translational modifications, impaired RNA-binding ability, aberrant liquid–liquid phase separation (LLPS), or oxidative stress conditions, which were well summarized and discussed in a review by Lye et al.^14^ Several studies have also suggested that early-stage TDP-43 oligomers form as intermediate species in the pathway to TDP-43 aggregation and inclusion formation.^15−17^

Although an increasing number of studies indicate that TDP-43 oligomers help regulate the function of TDP-43 in health and disease, it is unclear how the sequence and structural differences of these oligomers drive their formation, toxicity, or ability to seed TDP-43 aggregates. This is largely because isolating TDP-43 oligomers from brain tissues or cells is challenging due to their instability and heterogeneity. Consequently, obtaining sufficient quantities of TDP-43 oligomers for biophysical and biological studies is challenging. Furthermore, no efficient methods are available for producing purified full-length TDP-43 oligomers in vitro, thus precluding studies to investigate their structural, functional and toxic properties.

Different experimental approaches have been developed to decipher the role of TDP-43 oligomerization in the pathogenesis of ALS, FTLD, and other NDDs. For example, advances in optogenetic tools have enabled researchers to induce and visualize TDP-43 oligomerization dynamically in vitro and in vivo.^18,19^ In another chemogenetic method, the dimerization domain of a second protein is adopted as the clustering module in TDP-43 NTD, and homodimerization can be induced by a small-molecule ligand; as a result, TDP-43 NTD dimerization/oligomerization can be selectively induced.^20^ Through these tools and methods, researchers investigated the mechanisms underlying the LLPS, oligomerization and cytoplasmic mislocalization and aggregation of TDP-43 in ALS. However, in these model systems, the initial oligomerization events are not driven by TDP-43 but instead by other proteins fused to TDP-43, e.g., CRY2 multimerization tag and FKBP dimer domains.

In this work, we present a new method for producing pure preparations of unmodified and small-molecule-induced full-length TDP-43 oligomers. We show that in the presence of selected small molecules, such as EGCG, dopamine, and 4-HNE, TDP-43 is rapidly converted into stable oligomers that can be easily isolated using size-exclusion chromatography (SEC). Compared to the unmodified species, the modified oligomers (EGCG-O, DOPA-O, and HNE-O) showed different size distributions and morphologies. Interestingly, all four types of TDP-43 oligomers retained their RNA-binding ability in vitro and demonstrated only mild cytotoxicity to primary neurons. Furthermore, all four oligomer preparations could not seed the aggregation of the TDP-43 aggregation-prone amyloid core peptides (279–360). The ability to access these oligomers represents an important advance toward elucidating the role of TDP-43 in health and disease. Furthermore, our studies show that small-molecule stabilization of functional TDP-43 oligomers could be a promising strategy to inhibit aberrant fibrillization and the formation of pathological inclusions, although further studies are needed to test this hypothesis.

## Results

### Full-Length TDP-43 Forms Heterogeneous and Fibrillization-Resistant Oligomers

**Figure 1:**
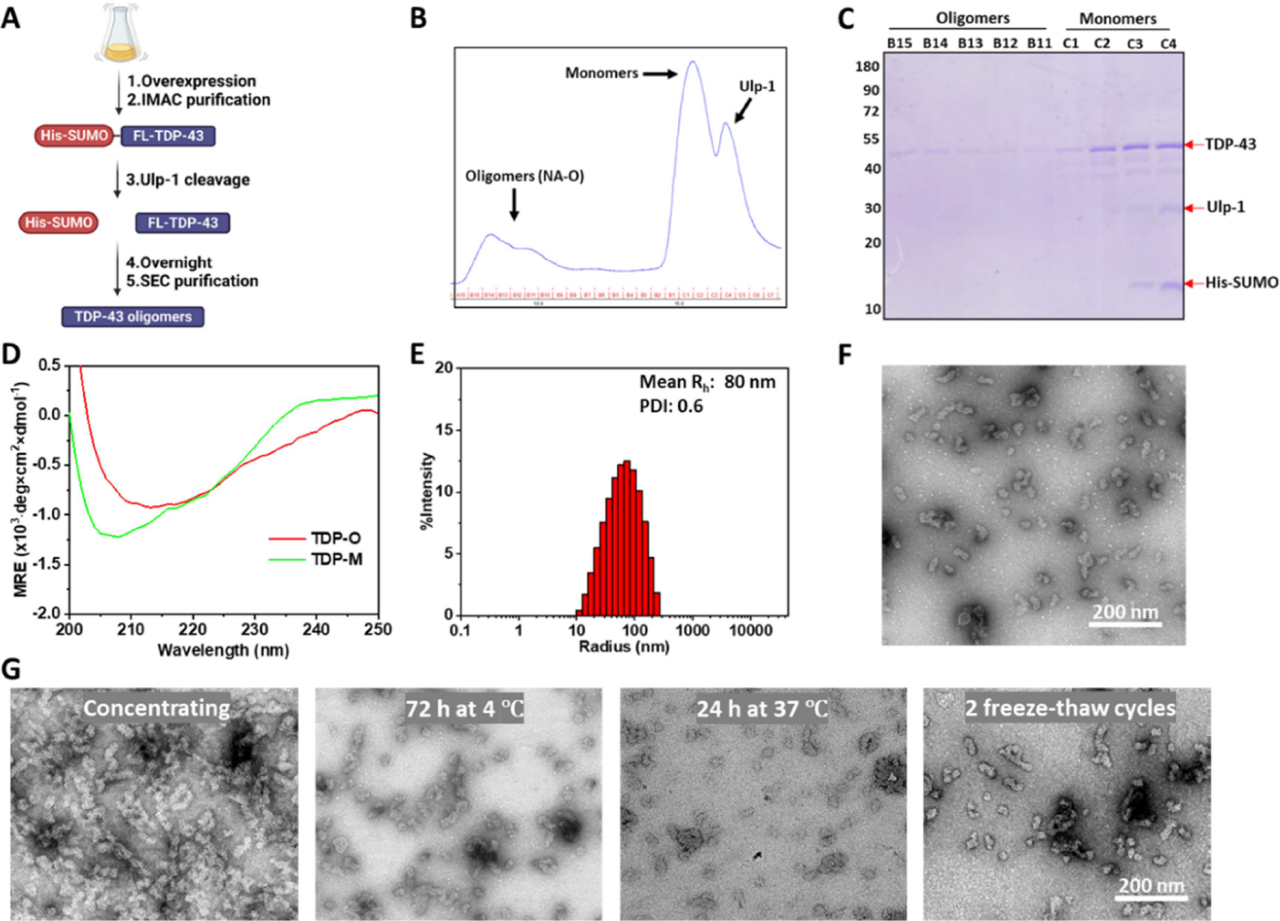
Generation and purification of native full-length TDP-43 oligomers. (A) Two-step protocol for generating TDP-43 native oligomers from His-SUMO-TDP-43. (B) Isolation of full-length oligomers using SEC-based purification after His-SUMO tag cleavage. (C) SDS–PAGE analysis of fractions corresponding to TDP-43 oligomer and monomer from the SEC purification. (D) Far-ultraviolet CD analysis of full-length TDP-43 monomers and oligomers. (E) Size-distribution analysis of TDP-43 oligomers by DLS. Mean *R*_h_: average hydrodynamic radius. PDI: polydispersity index. (F) TEM analysis of TDP-43 oligomers from fraction B13. (G) TEM image of concentrated native oligomers (30 μM) and native oligomers after 72 h of incubation at 4 °C, 24 h of incubation at 37 °C, or two freeze–thaw cycles. Scale bar: 200 nm.

Previously, our laboratory described an efficient protocol to produce full-length TDP-43 in *E. coli*.^21^ Briefly, TDP-43 was firstly expressed as a NTD-tagged His-SUMO fusion protein and purified by a HisTrap column. After the His-SUMO fusion tag was removed using ubiquitin-like-specific protease 1 (Ulp-1) overnight, TDP-43 rapidly formed oligomers that could be separated from monomeric TDP-43 by SEC (Figure 1A). As shown in Figure 1B, immediately after His-SUMO cleavage, the TDP-43 protein exists predominantly as monomers but in equilibrium with oligomeric species that elute in the void volume (7–10 mL). SDS–PAGE and mass spectrometry analysis confirmed that the oligomer-containing fractions were free of the fusion His-SUMO tag (Figure 1C and Figure S1B,C). Circular dichroism analysis revealed broad and redshifted minima, indicating a secondary structure transition from TDP-43 monomers to β-sheet-rich oligomers (Figure 1D). Dynamic light scattering (DLS) analysis showed that the purified TDP-43 oligomers (NA-O) had an average hydrodynamic radius (*R*_h_) of 80 nm, with a broad size range from approximately 10 to 200 nm (Figure 1E). In addition, the polydispersity index (PDI) which describes the degree of heterogeneity of a distribution^22^ was reported as 0.6, also suggesting a significant non-uniformity of these oligomers. TEM analysis demonstrated that there were two main populations of oligomeric species, with sizes of approximately 10 nm and 25–30 nm, respectively. In addition to globular and elongated oligomers, smaller homogeneous oligomers were prominently present in the background (Figure 1F). However, these two main species could not be separated using secondary SEC purification.

To explore the feasibility of using these oligomer preparations for biophysical and biological studies, we first assessed their stability under various storage conditions and at different concentrations. The concentration of the oligomers that we directly obtained from SEC purification was in the high nanomolar range. Therefore, the eluted fractions were pooled and concentrated to as high as 30 μM. TEM analysis showed that the concentrated oligomers retained their structure and morphological properties and did not aggregate into fibrils, although they were packed more closely with each other (Figure 1G). These observations suggest that monomers are necessary for the transition from oligomers to fibrils. Next, we subjected the oligomers to freeze–thaw cycles using liquid nitrogen or direct incubation at different temperatures, such as 4 °C and 37 °C. As shown in Figure 1G, these oligomers were stable at 4 °C for 72 h. However, the amount of the 10 nm oligomeric species was reduced significantly after incubation at 37 °C for 24 h or after 2 freeze–thaw cycles. Interestingly, the size of the high-molecular-weight oligomers remained unchanged. Taken together, the stability studies showed that recombinant native TDP-43 formed heterogeneous oligomers in vitro and suggested that the smaller oligomers may serve as building blocks for the high-order species; however, both species were resistant to fibrillar aggregation.

### Native TDP-43 Oligomers Maintain the Ability To Bind to TG-Rich Oligonucleotides and Do Not Possess Seeding Activity in Vitro

**Figure 2:**
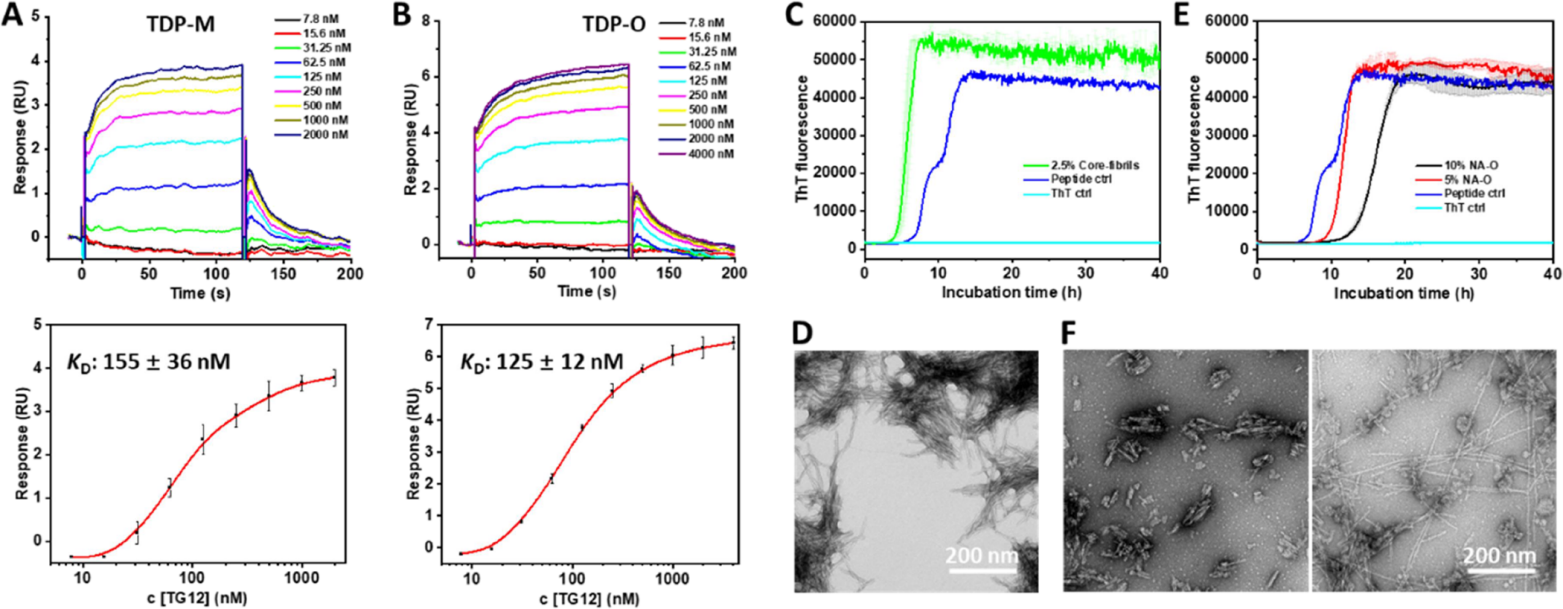
Binding and seeding studies of native TDP-43 oligomers in vitro. SPR binding assays between TG12 and TDP-43 monomer (A) or oligomer (B). (C) ThT kinetics of TDP-43 core peptide 279–360 in the presence and absence of its fibrillar seeds. (D) TEM image of TDP-43 core peptide aggregation with its fibrillar seeds. (E) ThT kinetics of TDP-43 core peptide in the presence of the full-length native oligomer seeds. (F) TEM images of peptide aggregation with 10% oligomer seeds. Scale bar: 200 nm.

One of the functional properties of TDP-43 is its ability to specifically bind several TG/UG-rich oligonucleotides, such as TG12, through its RNA-binding domains,^23,24^ and these interactions have been reported to stabilize the proteins and inhibit their aggregation.^25−27^ To determine whether the TDP-43 oligomers retain the ability to bind these ligands, we compared the binding affinity of these ligands to TDP-43 monomers and oligomers. Figure 2A shows a *K*_D_ value of 155 ± 36 nM between TG12 and monomeric TDP-43 (TDP-M), measured by surface plasmon resonance (SPR), which is comparable to recently reported values using different methods.^23,28,29^ Surprisingly, TG12 binds to TDP-43 oligomers with a similar binding affinity (125 ± 12 nM) (Figure 2B). Collectively, these results indicated that our oligomers may retain some of the functional properties of TDP-43, namely its ability to bind some of its ligands.

Several studies have suggested that the oligomeric forms of several amyloid proteins play central roles in mediating amyloid toxicity and cell-to-cell propagation of protein aggregates; this process is strongly dependent on their seeding activity, i.e., the ability to induce protein misfolding and fibril formation.^30−31,32^ Therefore, we next investigated the seeding activity of TDP-43 oligomers. The full-length TDP-43 aggregates and fibrils do not bind to thioflavin T (ThT) because their amyloid core is buried and covered by the RNA-binding domains; thus, we investigated the ability of TDP-43 oligomers to seed the aggregation-prone core peptide corresponding to residues 279–360 as described previously.^21^ This TDP-43 fragment forms highly ordered fibrils that bind to ThT, and its aggregation is accelerated by the addition of TDP-43 (279–360) fibrillar seeds (Figure 2C,D and Figure S2). In contrast, the addition of TDP-43 oligomers led to a longer lag-phase time, suggesting that the oligomers cannot seed the aggregation of TDP-43 (279–360) and may slow down TDP-43 aggregation, possibly through recruiting free monomers (Figure 2E). Interestingly, TEM analysis of these samples indicated that the presence of the oligomers resulted in the formation of fibrils with distinct morphologies (Figure 2F), in addition to possible amorphous aggregates. In addition, some peptides formed amorphous aggregates during the process. Altogether, our results revealed that the native unmodified TDP-43 oligomers possessed similar binding affinity to TDP-43 natural oligonucleotide ligands and could not induce the aggregation of its amyloid core peptides.

### TDP-43 Oligomerization Can Be Enhanced in the Presence of Selected Small Molecules

Although full-length TDP-43 readily forms oligomers, they do not accumulate in high amounts and rapidly convert to fibrils in the presence of TDP-43 monomers. This property precludes efforts to scale up their production and limits the number and type of studies that can be performed to investigate their biophysical, functional, and pathogenic properties. Therefore, we sought to identify conditions that enhance TDP-43 oligomer formation and/or favor the formation of stable oligomers. Previous studies have identified several polyphenol compounds (i.e., EGCG and dopamine) that can induce the oligomerization of several proteins (i.e., Aβ, α-synuclein, Tau) and inhibit their fibrillization.^33−35^ In addition, some reactive compounds, such as 4-hydroxy-2-nonenal (4-HNE), were shown to induce the formation of covalently modified and stable α-synuclein oligomers.^36,37^ Therefore, we sought to investigate whether these compounds could also induce TDP-43 oligomerization.

**Figure 3:**
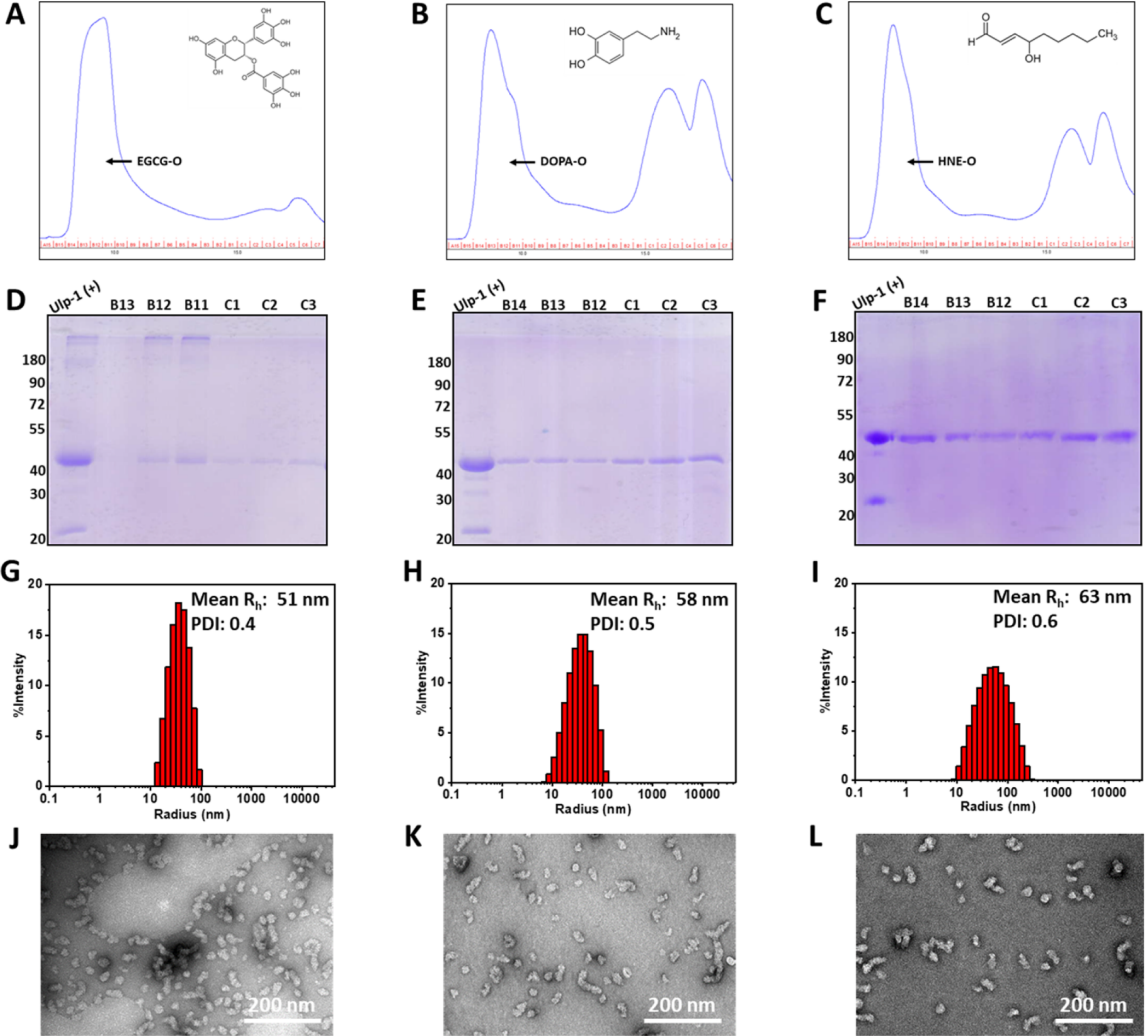
Production and biophysical characterization of chemically induced TDP-43 oligomers. (A–C) Isolation of EGCG/dopamine/4-HNE-induced oligomers using SEC purification. (D–F) SDS–PAGE analysis of fractions from SEC purification. DLS analysis (G–I) and TEM images (J–L) of these oligomers were collected from the void volume of SEC. Mean *R*_h_: average hydrodynamic radius. PDI: polydispersity index. Scale bar: 200 nm.

His-SUMO-fused TDP-43 was co-incubated with each compound at a ratio of 1:20 in the presence of Ulp-1 to initiate the cleavage and release of the native TDP-43 protein (overnight at 4 °C). The TDP-43 oligomers were then separated by SEC. As shown in Figure 3A, pretreatment with EGCG resulted in the conversion of >90% of nascent TDP-43 monomers into oligomers, as evidenced by the disappearance of monomers (approximately 15–17 mL). Similarly, pretreatment with dopamine and 4-HNE also induced high conversion of monomers to oligomers, although less efficiently (30% and 50%, respectively) than EGCG (Figure 3B,C). It has been reported that the number of phenol groups is a key factor leading to their inhibitory activity against aggregation.^38^ Therefore, the presence of more phenol groups in EGCG could explain their increased activity toward promoting TDP-43 oligomerization.

Interestingly, the oligomers formed in the presence of EGCG exhibited higher resistance to sodium dodecyl sulfate (SDS), as evidenced by the accumulation of a high molecular weight band representing TDP-43 aggregates in the stacking gel (Figure 3D). In contrast, the oligomers formed in the presence of dopamine and 4-HNE were SDS-sensitive, as TDP-43 in the void volume peak ran primarily as a single band with an apparent molecular weight corresponding to that of the monomer (Figure 3E,F). Furthermore, we compared the hydrodynamic size of these oligomers by DLS. As shown in Figure 3G–I, the hydrodynamic radii for EGCG-O, DOPA-O, and HNE-O were approximately 51 nm, 58 nm, and 63 nm, respectively. The polydispersity indexes of these species were 0.4–0.6, suggesting that these oligomers were also quite heterogeneous in size as the native oligomers. In addition, TEM analysis further confirmed that these oligomers appear less heterogeneous than the unmodified oligomers since they predominantly were found in one size population, which was approximately 25–30 nm in diameter of various lengths; furthermore, smaller species at 10 nm were not observed, as previously found in NA-O samples (Figure 3J–L). CD analysis of these oligomers did not show much difference in the secondary structure composition, although the secondary structure prediction using a Web server (BeStSel: https://bestsel.elte.hu/index.php)^39^ based on the CD data indicated that the β-sheet percentage was slightly increased for DOPA-O and HNE-O and slightly decreased for NA-O and EGCG-O compared to monomeric TDP-43 (Figure S3). Finally, the stability of these modified oligomers was also investigated. The results in Figure S4 show that these oligomers are as stable as NA-O, as their morphology did not undergo significant changes under different handling and storage conditions (concentrating and freeze-and-thaw cycles) and temperatures (4 °C for 72 h and 37 °C for 24 h).

To determine the mechanism by which these compounds induce TDP-43 oligomer formation, these oligomers were denatured and analyzed by mass spectrometry. We found that all chemically induced oligomers were covalently modified, as shown in Figure S5. Apart from the mass of full-length TDP-43, we observed the presence of additional peaks of larger mass, indicating that the protein underwent chemical modifications. The mass increments observed for the EGCG-O and DOPA-O samples corresponded to the addition of one molecule (+471 and +147, respectively) of the corresponding compounds. In the case of HNE-O, two significant modifications (+152 and +309) can be found, which corresponded to mass increments for one and two molecules.

Altogether, these results suggest that the chemical modification of TDP-43 monomers could serve as a mechanism for inducing TDP-43 misfolding and oligomerization. Furthermore, the chemically induced oligomers are stable and thus suitable for more mechanistic studies on TDP-43 aggregation.

### All Chemically Induced TDP-43 Oligomers Exhibit Similar Binding Affinity to TG12 and Do Not Seed the Aggregation of TDP-43 (279-360)

**Figure 4:**
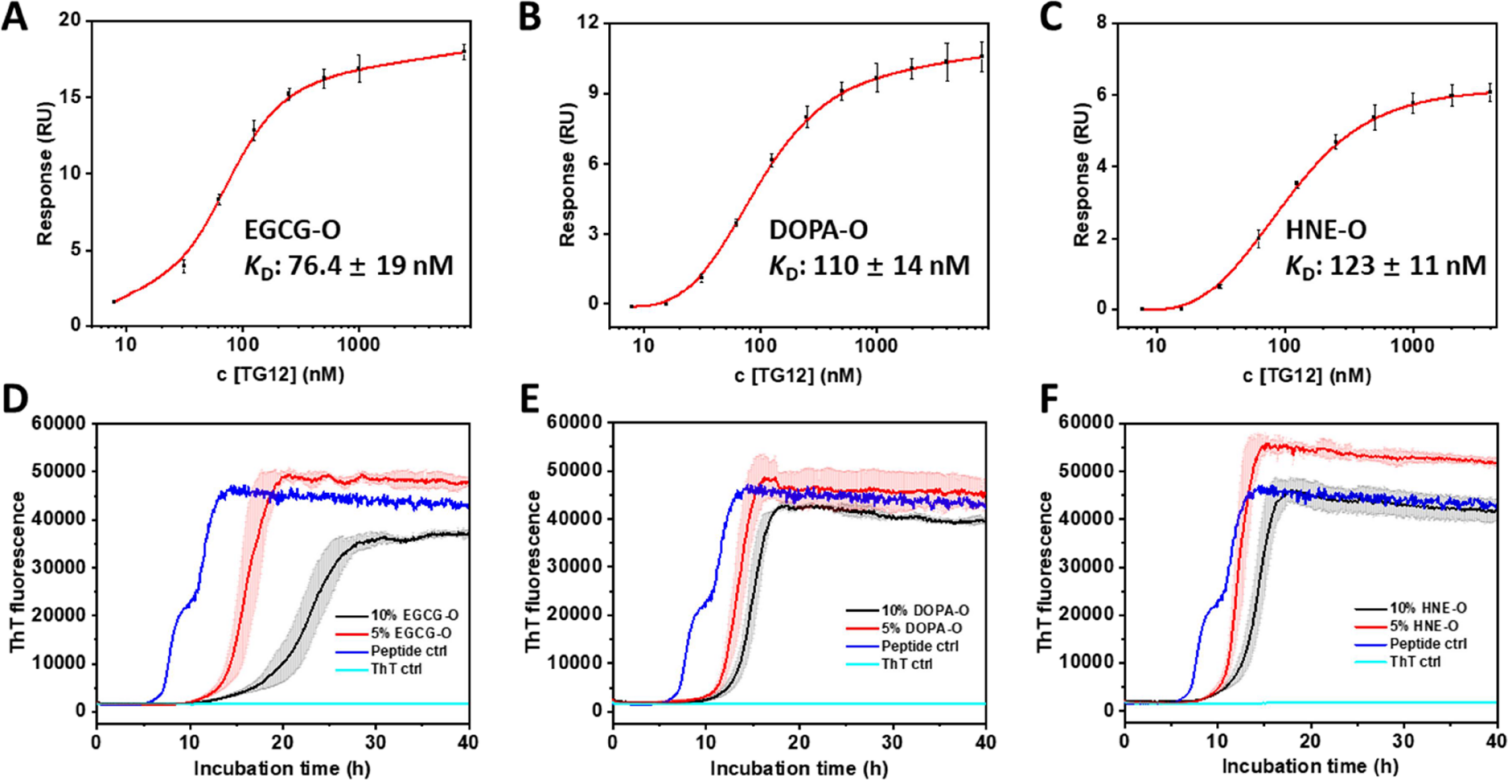
Binding and seeding studies of chemically induced TDP-43 oligomers. (A–C) SPR binding affinities between oligomers (EGCG-O, DOPA-O, or HNE-O) and the TG12 ligand. (D–F) ThT kinetics-based aggregation of TDP-43 core peptide 279–360 in the presence of different full-length oligomers. The experiments in D–F were performed simultaneously, and for clarity, the data for the control peptide is included in each graph.

Next, we investigated the ability of the EGCG-O, DOPA-O, and HNE-O TDP-43 oligomers to bind to their cognate UG/TG-rich oligonucleotide ligands. Our results demonstrated that the binding affinities measured by SPR assay (Figure 4A–C) were slightly enhanced for the modified oligomers compared to the unmodified oligomers, with the following ranking: EGCG-O > DOPA-O > HNE-O ≈ NA-O. Interestingly, the binding affinity seems to correlate with the hydrodynamic radius measured by DLS in Figure 3G–I, with smaller size oligomers exhibiting higher binding affinity. However, the factors responsible for this difference in binding affinity were not further explored.

Furthermore, the seeding ability of these chemically induced oligomers was evaluated under the same conditions as the unmodified oligomers through the ThT kinetics-based aggregation assay. As shown in Figure 4D, the aggregation process of TDP-43 core peptide 279–360 was dramatically delayed when the peptide was co-incubated with EGCG-O. The inhibitory effect of 5% EGCG-O treatment was almost comparable to that of 10% NA-O. For DOPA-O and HNE-O, the aggregation-inhibiting effect was weaker than that of the EGCG-O seeds and was even weaker than that of NA-O (Figure 4E,F and Figure S6). The ranking in terms of the lag phase time extension was as follows: EGCG-O > NA-O > DOPA-O ≥ HNE-O. Collectively, none of these TDP-43 oligomers can seed or accelerate the aggregation of TDP-43 core peptides.

### Limited Proteolysis of TDP-43 Oligomers Does Not Influence Their Seeding Activity in Vitro

A recent study from our laboratory showed that full-length TDP-43 forms highly organized filaments driven by the C-terminal domain corresponding to residues 279–360. The fibrillar core formed by this domain is completely buried by the flanking ordered domains, which explains why fibrils derived from the full-length protein do not exhibit seeding activity in vitro or in cells. Proteinase K (ProK)-mediated proteolytic cleavage of the full-length fibrils or brain-derived fibrils leads to exposure of the amyloid core and converts them into seeding competent fibrils; i.e., they induce seeding of TDP-43 (279–360).^40^ Therefore, the lack of seeding activity by the oligomers could also be due to the inaccessibility of the amyloid core, which was buried by the flanking RNA binding domains.

**Figure 5:**
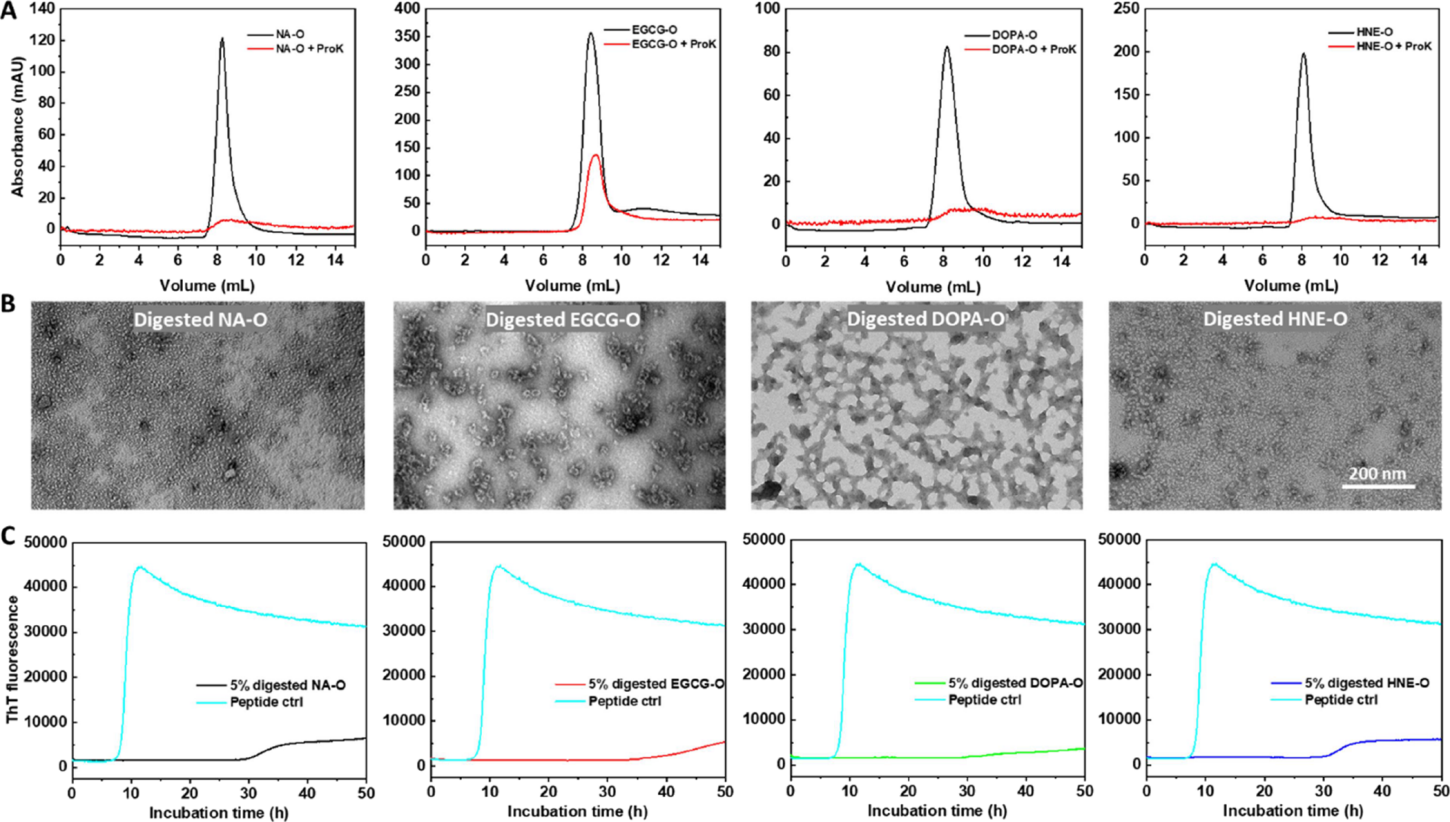
Seeding studies of digested TDP-43 oligomers mediated by ProK proteolysis on the aggregation of the core peptide 279–360. (A) SEC purification of TDP-43 oligomers in the absence and presence of ProK pretreatment before sample injection (absorbance: 280 nm). (B) ProK-digested TDP-43 oligomers collected from SEC purification. Scale bar: 200 nm. (C) ThT kinetics of TDP-43 core peptide aggregation in the presence of digested oligomers.

To test this hypothesis, we exposed the newly formed oligomers to ProK and then repurified the remaining protein species by SEC. As shown in Figure 5A, the oligomer peaks dropped dramatically when samples were pretreated with ProK compared to the control samples without predigestion. TEM analysis of the treated oligomers, which eluted in the void volume peaks, revealed the presence of smaller oligomers in all samples, suggesting that digestion of the original oligomers was limited (Figure 5B). These oligomers were concentrated and then used to seed the aggregation of TDP-43 (279–360). Figure 5C shows that the digested oligomers did not induce seeding activity but rather induced a significant delay in the aggregation of the peptides. Noticeably, the inhibitory effect of the digested materials was much stronger than that of the intact oligomers (Figures 2E and 4D–F**)**. Taken together, the results indicate that neither unmodified nor chemically modified TDP-43 oligomers are seeding competent in vitro.

### TDP-43 Oligomers Exhibited No or Very Mild Toxicity in Primary Cells

It has been suggested that oligomeric forms of aggregation-prone proteins may be a primary toxic species that contributes to neurodegeneration in many NDDs. Compared to stable fibrils, oligomers are smaller in size, more soluble, and easier to diffuse.^31,41,42^ Therefore, we investigated the cytotoxic properties of TDP-43 oligomers in mice primary neurons.

Previous studies estimated that the physiological concentration of TDP-43 within a cell was approximately 4 × 10^6^ (2–3 μM).^43^ Therefore, we investigated the toxicity of our oligomer preparations after addition to culture media of hippocampal or cortical neurons at 2 μM. We monitored cell death using two methods, lactate dehydrogenase (LDH) and NeuN counting.

**Figure 6:**
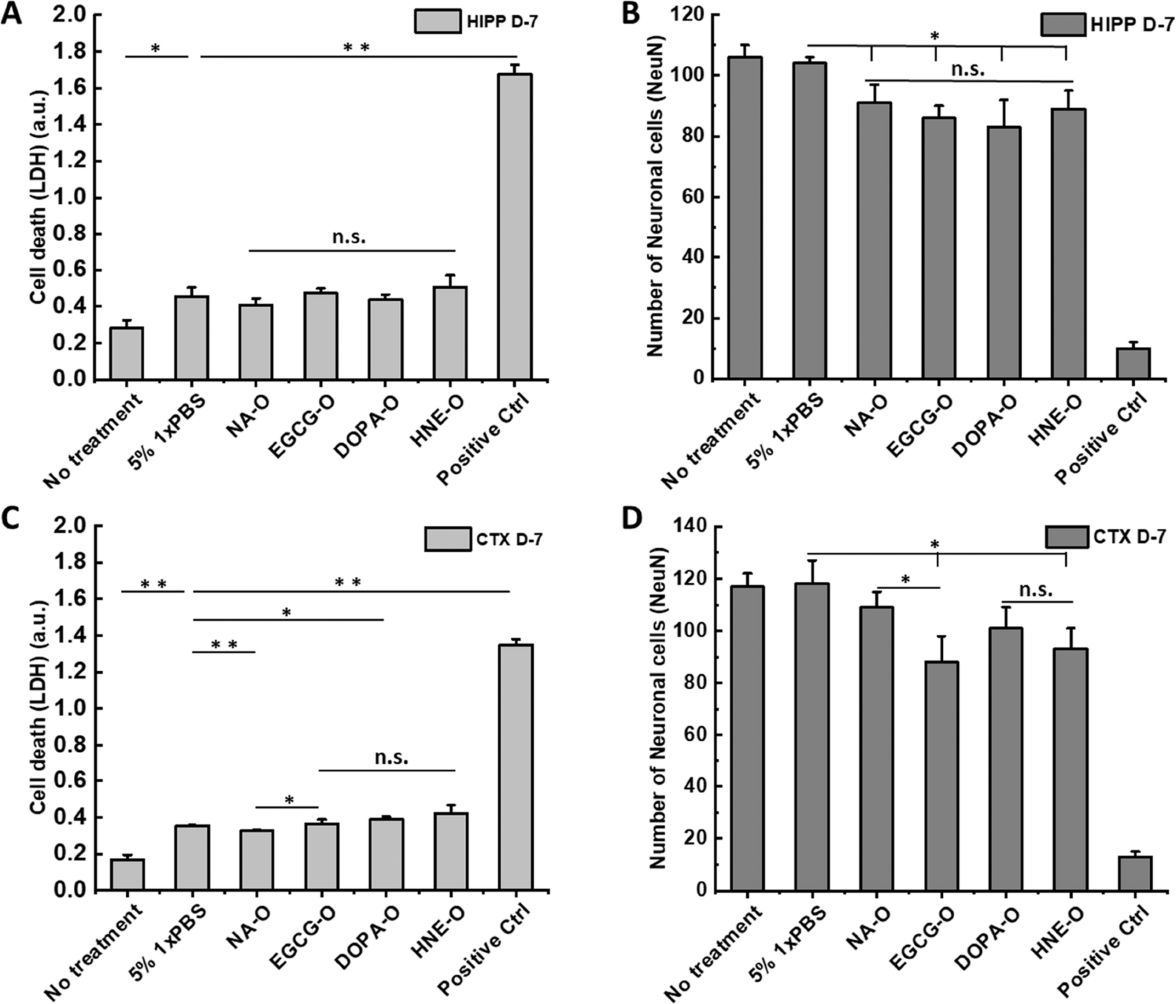
Cytotoxicity studies of different TDP-43 oligomers. (A) LDH assays of oligomers in primary hippocampal neurons. (B) Hippocampal neuron count based on nuclear staining with an anti-NeuN antibody. (C) LDH assays of oligomers in primary cortex neurons. (D) Cortical neuron count based on nuclear staining with the NeuN antibody (*n* = 3, mean ± sem). Positive control: cells treated with 1% Triton-100 during cell culture. Unpaired *t* test was used to compare individual type of oligomers with the negative buffer control, and ANOVA test was used to analyze toxicity data among different oligomers (* = *p* < 0.05 over 1× PBS buffer control or among oligomers, ** = *p* < 0.001 over 1× PBS buffer control, n.s. = no significance).

Both assays showed that all four types of oligomers induced mild or negligible toxicity after 7 days of treatment in primary neurons. However, the difference among these oligomers was not significant in hippocampal neurons (Figure 6A,B). The neuronal toxicity of oligomers was only significant compared to the non-treated control in the LDH assay. Quantification of the remaining healthy neurons showed a similar toxicity profile (Figure 6B), but these oligomers were also slightly toxic compared to the PBS buffer control in terms of NeuN count. The morphology of neurons remained almost the same, as shown in Figure S7A. In cortical neurons, the unmodified oligomers NA-O manifested slightly reduced toxicity in the LDH assay, but there were no significant differences among the other three types of TDP-43 oligomers (Figure 6C). In terms of NeuN count, the toxicity profile looked similar to that of the LDH assay except that EGCG-O induced slightly more neuronal death than NA-O (Figure 6D and Figure S7B). A previous study by Fang et al reported dose-dependent cellular toxicity of TDP-43 oligomers and showed an approximately 20% reduction in cell viability at the highest concentration (0.5 μM).^28^ However, in this study, the authors used a TDP-43 construct with an N-terminal histidine tag and obtained TDP-43 oligomers that exhibit structural and morphological features that are distinct from those used in our studies. Further studies are needed to more systematically investigate how the presence of oligomers influences cellular/neuronal dysfunction and not only cell death. The methods we describe here pave the way for conducting these studies.

## Discussion

Although oligomeric intermediates on the pathway to amyloid fibril formation of several proteins, such Aβ, Tau, and α-synuclein, continue to gain attention as central players in the pathogenesis of neurodegenerative diseases,^31,32^ very little is known about the biochemical, biophysical, and pathogenic properties of TDP-43 oligomers, which are essential for elucidating the role of TDP-43 oligomers in health and disease. To address this knowledge gap, efficient protocols must be developed to purify and/or prepare different types of TDP-43 oligomers for further mechanistic studies.

In this study, we describe methods for generating pure preparations of WT native TDP-43 oligomers using the full-length recombinant protein. However, the overall yield of oligomers was extremely low because of the high propensity of full-length TDP-43 to aggregate into insoluble fibrils once the His-SUMO tag was removed. We explored another approach that was previously successful for other amyloid-forming proteins, namely, the stabilization of oligomers through small-molecule interactions and/or small molecule-induced chemical modifications.^44,45^ For this study, we selected the following compounds, EGCG, dopamine, and 4-HNE, which were shown to induce the formation and accumulation of α-synuclein oligomers of distinct size and morphology distribution.^46,47^

As expected, all three compounds interacted with TDP-43 and increased the efficiency of its oligomerization, which could be easily purified from monomers by SEC. Compared to the unmodified oligomers, these chemically induced oligomeric species were all structurally distinct. Their lower hydrodynamic radii may reflect changes in the compactness of the TDP-43 oligomers or that these oligomers were composed of a smaller number of monomers. Interestingly, all three types of oligomer preparations maintained the ability to bind TDP-43 ligands, the TG-rich oligonucleotides. In addition, the three TDP-43 oligomer preparations could not seed the aggregation of the C-terminal fragment that comprises the amyloid core fibrils and exerted only mild neuronal toxicity. In our study, cell death was used as the main readout for toxicity; thus, oligomer-induced cellular dysfunctions that did not cause cell death within the timeframe of our experiments were not considered. Therefore, future studies should examine the effect of oligomers on RNA metabolism and changes in gene transcription and the TDP-43 interactome upon internalization of oligomers.

Furthermore, we understand that the toxicity of in vitro generated TDP-43 oligomers could differ from that of native oligomers, and the structure and toxic properties of TDP-43 oligomers may be further modulated by post-translational modifications (i.g., phosphorylation, truncations), mutations, and/or interactions with other cofactors.^14^ Prior to our studies, Fang et al also reported on a method for producing full-length TDP-43 oligomers. In contrast to our TDP-43 oligomer preparations, these oligomers exhibited distinct morphology, showed decreased binding affinity to natural oligonucleotide ligands, and manifested toxicity to neuronal cells in low concentrations. It was also shown that these oligomers were immunoreactive to a conformation-dependent anti-amyloid oligomer-specific antibody (A11),^48^ indicating that they were amyloid-like, which is different from our preparations. We speculate that these differences could be due to the fact that the TDP-43 construct they used contained a histidine tag with additional 23 amino acid, whereas we used native TDP-43.^28^

The oligomer preparation protocols we present pave the way for testing these hypotheses and call for further studies to investigate how known TDP-43 ligands influence the functions, dynamics, and structural landscape of TDP-43 oligomers. Our ability to generate large quantities of these oligomers in vitro should also enable the generation of new tools, e.g., antibodies, and assays for detecting, monitoring, and quantifying TDP-43 oligomer formation in cells, which is essential for elucidating their role in health and disease. In parallel, it is crucial to develop and optimize reproducible methods to isolate native TDP-43 oligomers. In-depth characterization of these oligomers could inform future efforts to develop efficient protocols that reproduce their features under controlled conditions in vitro. Importantly, our work also demonstrates the feasibility of identifying small molecules that inhibit TDP-43 fibril formation through the stabilization of TDP-43 oligomers without potentially altering their functional properties.

## Methods

### Preparation of HIS-SUMO Fusion TDP-43

The N-terminal His-SUMO fusion protein was prepared following a protocol previously developed in our laboratory.^40^ Briefly, the plasmid was transformed and overexpressed in *E. coli* strain BER2566. A 3 L bacterial culture was harvested at 4000 rpm for 15 min at 4 °C. The pellets were then resuspended in 120 mL of lysis buffer on ice (30 mM Tris, 15 mM imidazole, 0.5 M NaCl, 10% v/v glycerol, 1 mM DTT, pH 8.0). PMSF (2 mM) and 3 tablets of complete protease inhibitors were quickly added and dissolved with stirring. Cells were lysed by sonication (5 min, pulse on 10 s, pulse off 10 s, 70% amplitude). Supernatants were separated by centrifugation (45 min, 14 000 rpm, 4 °C), followed by the addition of 12 μL of benzonase nuclease HC (Novagen) and mild stirring at room temperature for 30 min. The crude sample was filtered (0.45 μM syringe filter membrane) and loaded quickly into a 20 mL preequilibrated His-trap affinity column (GE Healthcare). After that, weakly bound and tag-free proteins were washed off by 5 column volumes of buffer A (30 mM Tris, 15 mM imidazole, 0.5 M NaCl, 10% v/v glycerol, 1 mM DTT, pH 8.0) at a flow rate of 2 mL/min. Imidazole gradient elution with buffer B (30 mM Tris, 500 mM imidazole, 0.5 M NaCl, 10% v/v glycerol, 1 mM DTT, pH 8.0) from 0 to 40% and then to 100% was carried out to collect the fusion protein. Purified fusion TDP-43 was identified using SDS–PAGE (15% polyacrylamide gels), ESI-MS, and analytical C4 reversed-phase ultrahigh-performance liquid chromatography (UPLC). The theoretical molecular weight is 57775.2 Da.

### Preparation of TDP-43 Oligomers

Oligomers were prepared by SEC. The freshly prepared fusion protein (1 mL) was cleaved overnight at 4 °C using Ulp-1 (prepared in-house) and checked by UPLC and SDS–PAGE. Subsequently, the cleaved sample was briefly centrifuged to remove any preformed aggregates. Next, 1 mL of supernatant was injected into the SEC column (Superdex 200, 100/300 increase GL, Cytiva) and washed with the buffer (30 mM Tris, 300 mM NaCl, 1 mM DTT, pH 8.0) at 0.4 mL/min. Eluted samples (0.5 mL per fraction) were analyzed by SDS–PAGE, ESI-MS, and TEM to confirm the presence of full-length oligomers.

To prepare chemically induced oligomers, compounds (20 times excess) were co-incubated with fusion TDP-43 and Ulp-1 overnight, and other steps were the same. For the digested oligomeric species, protein samples that were cleaved overnight were treated with ProK (final concentration: 1 μg/mL) for 30 min at RT. The resulting samples were quickly injected into the SEC column for purification.

Oligomers were concentrated into desired concentrations using Amicon tubes (Amicon Ultra15 centrifugal filter units 50 kD, Merck Millipore) for other purposes.

### ESI-MS

For His-SUMO-TDP-43 and full-length TDP-43 oligomers purified in Tris buffer (30 mM Tris, 300 mM NaCl, 1 mM DTT, pH 7.4), 15 μL of each sample (around 0.1 mg/mL) was taken and analyzed using electrospray ionization (ESI) in positive ion mode (3.5 kV ionization voltage) on an LTQ Orbitrap XL (Thermo Fisher), equipped with an Accela pump HPLC and a CTC ThermoPAL autosampler (Thermo). Proteins were separated on a 4.6 mm inner diameter × 75 mm length column, packed with 3.5 μm, 100 Å Symmetry C18 (Waters). The analysis was performed using a 10 min gradient at a flow rate of 300 μL/min with a mixture of 0.1% formic acid in 2% acetonitrile (mobile phase A) and 0.1% formic acid in 98% acetonitrile (mobile phase B). The mass spectrometer operated with a full scan range of 400–2000 *m*/*z* and a resolving power of 60 000 *m*/*z*. The spectra were processed in MagTran 1.03. The molecular weight of the unmodified full-length TDP-43 was found as 44 606 kDa, which correlated with the native protein (2-414, Met1 in the NTD of TDP-43 was cleaved with the His-SUMO-tag). The amino acid sequence of the final native protein is as follows: SEYIRVTEDENDEPIEIPSEDDGTVLLSTVTAQFPGACGLRYRNPVSQCMRGVRLVEGILHAPDAGWGNLVYVVNYPKDNKRKMDETDASSAVKVKRAVQKTSDLIVLGLPWKTTEQDLKEYFSTFGEVLMVQVKKDLKTGHSKGFGFVRFTEYETQVKVMSQRHMIDGRWCDCKLPNSKQSQDEPLRSRKVFVGRCTEDMTEDELREFFSQYGDVMDVFIPKPFRAFAFVTFADDQIAQSLCGEDLIIKGISVHISNAEPKHNSNRQLERSGRFGGNPGGFGNQGGFGNSRGGGAGLGNNQGSNMGGGMNFGAFSINPAMMAAAQAALQSSWGMMGMLASQQNQSGPSGNNQNQGNMQREPNQAFGSGNNSYSGSNSGAAIGWGSASNAGSGSGFNGGFGSSMDSKSSGWGM.

### Transmission Electron Microscopy (TEM)

Prior to sample loading, Formvar and carbon-coated 200 mesh containing copper EM grids (Electron Microscopy Sciences) were glow-discharged for 30 s at 20 mA using a PELCO easiGlow glow discharge cleaning system (TED PELLA, Inc.). Subsequently, 5 μL of samples were spotted onto the EM grids and waited for 5 min. TDP-43 oligomer samples were then carefully blotted out using the edge of filter papers and air-dried for 1 min. After that, the grids were washed 3 times with ultrapure water, followed by staining with 0.7% (w/v) uranyl formate solution. The grids were subsequently examined using a Tecnai Spirit BioTWIN electron microscope. The microscope was equipped with a LaB6 gun operated at an acceleration voltage of 80 kV, and images were captured using a 4K × 4K charge-coupled device camera (FEI Eagle).

### Dynamic Light Scattering (DLS)

DLS measurements of the hydrodynamic radius of TDP-43 oligomers were performed at RT using a DynaPro plate reader II (Wyatt Technology) with a 384-well black microplate with an optical transparent bottom (Corning, 3540). Thirty microliters of sample was loaded and measured in 5 s (5 acquisitions each) with autoattenuation. Each sample was repeated 3 times in parallel. The results were analyzed by dynamic software (v7.10.1.21).

### Far-UV Circular Dichroism (CD) Spectroscopy

CD spectra of TDP-43 samples (monomer and oligomers) were loaded in a quartz cuvette with a 1 mm path length and were collected using a Jasco J-815 CD spectrophotometer operated at 20 °C within 200–250 nm. The sample volume was 150 μL per sample. Data were acquired with the following parameters: data pitch, 0.2 nm; bandwidth, 1 nm; scanning speed, 50 nm/min; digital integration time, 2 s. The spectra of each sample were the average of 10 repeats followed by a binomial approximation. The processed spectra were obtained by subtracting the baseline signal (quartz cuvette) from the protein spectra with no further smoothing. The raw data were converted to mean residue ellipticity (θMRW) and plotted using Origin 2021 software. Secondary structure prediction was performed using an online Web server (BeStSel: https://bestsel.elte.hu/index.php) with the CD data.

### Surface Plasmon Resonance (SPR)

The interactions between TDP-43 samples and TG12 were analyzed using a Biacore 8K system (GE Healthcare, Uppsala, Sweden). Briefly, protein samples (30 μg/mL, pH 4.0 in NaAc) were immobilized on a sensor chip CM5 (series S) by amine coupling after chip surface activation using EDC and NHS (1:1). A gradient concentration of TG12 (7.8–8000 nM) in the running buffer (1× PBS + 0.02% v/v Tween-20) was injected as the analytes. The association and dissociation times were 120 s. Data were analyzed by Biacore Evaluation software using 1:1 binding model (version 8K) and Origin 2021.

### Preparation of Fibrillar Seeds of TDP-43 Core Peptide 279-360

The peptide was prepared according to our recent paper.^21^ Then, 150 μg of lyophilized TDP-43 core peptide 279–360 was disaggregated in 100 μL of HFIP/TFA (1:1) buffer at 30 °C for 30 min. The solution was evaporated under a mild nitrogen stream. The peptide film was dissolved in fibril-forming buffer (30 mM Tris, 100 mM NaCl, pH 7.4) to give a final concentration of 50 μM (2.5% DMSO, v/v). The sample was incubated without shaking at room temperature, followed by sonication (1 s on and 1 s off for 5 s at 40% amplitude; repeated with 20% amplitude) to obtain the fragmented fibrillar seed. The presence of fibrils was confirmed by TEM analysis.

### Thioflavin T (ThT)-Based Aggregation Assay

Lyophilized TDP-43 core peptide 279–360 was disaggregated as described above. For the aggregation assay, 300 μL of each sample was prepared with the following composition: 5 μM core peptide, 20 μM ThT, and 5% or 10% different oligomers (for digested oligomers, the final concentration was 0.25 μM). Oligomer buffer and ThT alone were used as negative controls, and 2.5% core peptide seeds were used as the positive control. Samples (95 μL) in triplicate were pipetted into polybase black 96-well plates with optical bottoms (Corning, 3615). The plate was sealed, and studies on ThT kinetics were carried out in a FLUOstar Omega plate reader (BMG Labtech) by recording the ThT fluorescence online every 300 s using a 440 nm excitation filter and a 480 nm emission filter set at 25 °C. The plate was shaken at 100 rpm for 5 s before each measurement. The total running time was 40 h, the aggregation curve of every sample shown is the average of 3 independent recordings, and error bars show the standard deviation of the measurements. At the end of the measurements, 5 μL samples were taken for TEM analysis.

### Primary Cell Culture

Primary hippocampal and cortical neurons were prepared from postnatal D0 pups of WT mice (C57BL/6JRj; Janvier, France). All procedures were approved by the Swiss Federal Veterinary Office (authorization numbers VD3392 and VD3694). Briefly, the cerebral hippocampi and the cortexes were isolated stereoscopically in Hanks’ balanced salt solution and digested by papain (20 U/mL; Sigma-Aldrich) for 30 min at 37 °C. After papain activity was inhibited using a trypsin inhibitor (Sigma-Aldrich), tissues were dissociated by mechanical trituration. Cells were finally resuspended in adhesion media (MEM; 10% horse serum, 30% glucose, l-glutamine, and penicillin–streptomycin; Life Technologies) and plated in 24- or 96-well plates previously treated with 0.1% (w/v) poly-l-lysine in water (Brunschwig, Switzerland) at a density of 300 000 cells/mL (for biochemistry analysis) or 250 000 cells/mL (for ICC/confocal microscopy analysis). After 3 h, the adhesion medium was removed and replaced with neurobasal medium (Life Technologies) containing B27 supplement (Life Technologies), l-glutamine, and penicillin–streptomycin (100 U/mL). Neurons were plated directly in neurobasal medium in black, clear-bottom, 96-well plates at a concentration of 200 000 cells/mL. Newly prepared primary cells were cultured at least one week before sample treatment.

### Cell Viability Assay

On the day of the treatment, different TDP-43 oligomers purified in 1× PBS (pH 7.4) were diluted to a final concentration of 2 μM with neurobasal media collected from wells containing plated neurons. The leftover neurobasal media in the wells was aspirated and replaced by media containing the diluted oligomers. Treated neurons were cultured for up to 7 days without any further media changes until the end of the treatment. Positive control: cells treated with 1% Triton-100 during cell culture. Negative controls: nontreated cells and cells treated with equal 1× PBS as the oligomer samples.

A cytotoxicity assay of TDP-43 oligomers was performed using a CytoTox-96 nonradioactive cytotoxicity assay kit (Promega, Switzerland), and the LDH released into culture supernatants was measured according to the manufacturer’s instructions. After the coupled enzymatic reaction was performed using the treated culture medium, the amount of the red formazan product (proportional to the number of damaged cells) was measured using an Infinite M200 Pro plate reader (Tecan) at a wavelength of 490 nm.

### NeuN Counting

Cell death was also quantified by the total count of cells. The remaining treated neurons in the 96-well plate were fixed at the indicated times in 4% PFA (para-formaldehyde) for 20 min at RT and then washed away using 1× PBS. Neurons were permeabilized and blocked in a 1× PBS (pH 7.4) solution composed of 0.1% Triton X-100 and 3% BSA for 30 min at RT. The cells were then stained using an antibody against the nuclear neuronal marker protein NeuN (1:2000) and an antibody against the microtubule-associated protein MAP2 (1:2000). The nucleus was counterstained with DAPI at 1:1000 (Sigma-Aldrich, Switzerland). Cells were washed 2 times in 1× PBS afterward, and the plate was then examined with a microscope (LSM 700 inverted, Zeiss) with a 20× objective and analyzed using ImageJ (U.S. NIH, Bethesda, MD, USA; RRID, SCR_003070).

Results were plotted using Origin 2021. Unpaired *t* test and ANOVA test were used for the statistical analysis.

## Supporting Information Available

Protein purification and fibrillar seed preparation, additional oligomer stability analysis, additional CD data, ESI-MS analysis of oligomers, additional ThT kinetics-based seeding assay, and cytotoxicity studies (PDF)
