# Supplementary material for: Recombinant Full-Length TDP-43 Oligomers Retain Their Ability to Bind RNAs, Are Not Toxic, and Do Not Seed TDP-43 Aggregation in Vitro
Source: ACS Chem Neurosci. 2023 Dec 20;15(1):193–204. doi: 10.1021/acschemneuro.3c00691 (PMC10767740; doi:10.1021/acschemneuro.3c00691)
Supplement: Supplementary file 1 — cn3c00691_si_001.pdf [file cn3c00691_si_001.pdf]

Supporting information

Recombinant full-length TDP-43 oligomers retain their ability to bind RNAs, are not toxic, and do not seed TDP-43 aggregation in vitro

Lixin Yang, Yllza Jasiqi, Hilal Lashuel*

Laboratory of Molecular and Chemical Biology of Neurodegeneration, Institute of Bioengineering, EPFL, Switzerland

* To whom correspondence should be addressed: Laboratory of Molecular and Chemical Biology of Neurodegeneration, Institute of Bioengineering, Ecole Polytechnique Fédérale de Lausanne, 1015 Lausanne. Tel: +41216939691, Fax: +41216939665, Email: hilal.lashuel@epfl.ch.

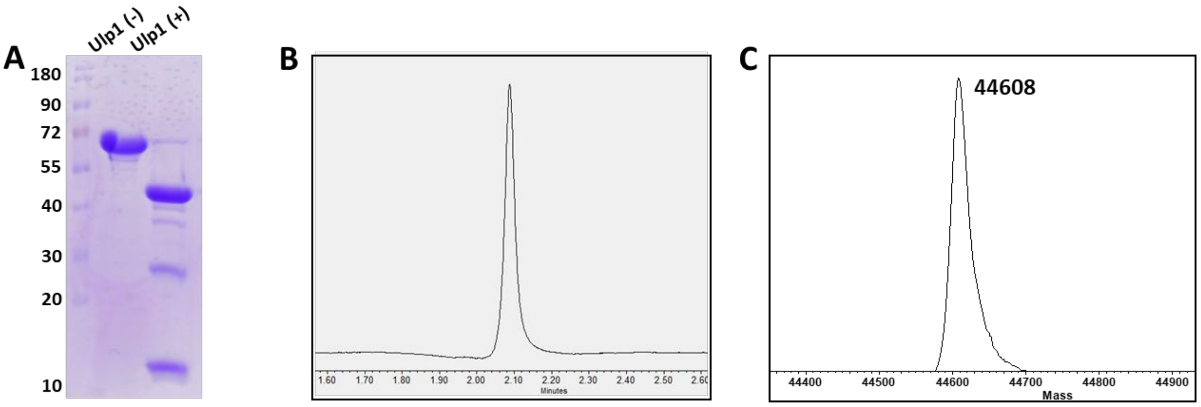

Figure S1. Purification and identification of full-length TDP-43 monomers and oligomers. (A) SDS–PAGE analysis of fusion TDP-43 cleavage by Ulp-1. (B) + (C) UPLC and ESI-MS analysis of oligomeric TDP-43 from SEC purification.

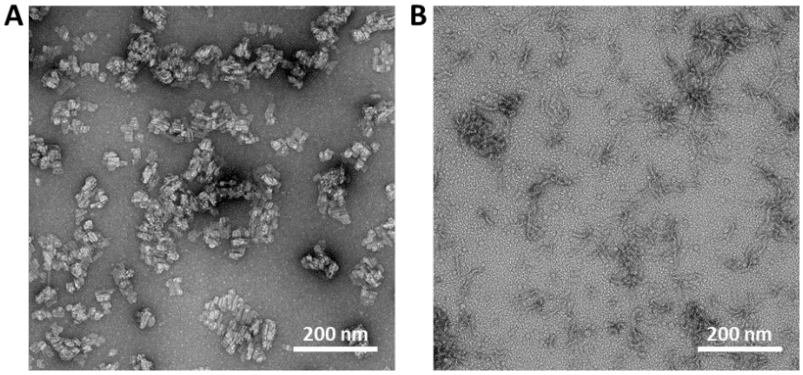

Figure S2. Preparation of fibrillar seeds of TDP-43 core peptide 279-360. Fibrils formed before (A) and after (B) sonication. Scale bar: 200 nm.

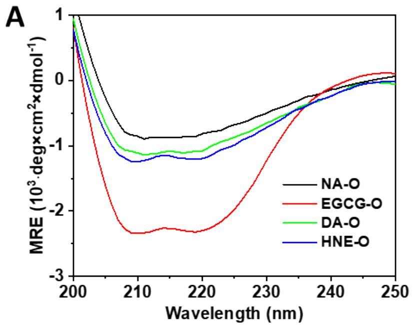

B

	α-helix	β-sheet	Turn	Others
TDP-M	13.8%	24.5%	12.4%	49.3%
NA-O	8.7%	19.2%	15.5%	56.6%
EGCG-O	13.2%	21.1%	13.6%	52.1%
DOPA-O	11.7%	33.9%	13.2%	41.2%
HNE-O	12.8%	30.6%	12.6%	44.0%

Figure S3. (A) CD analysis of four types of TDP-43 oligomers. (B) Secondary structure prediction of TDP-43 monomers and oligomers based on CD analysis.

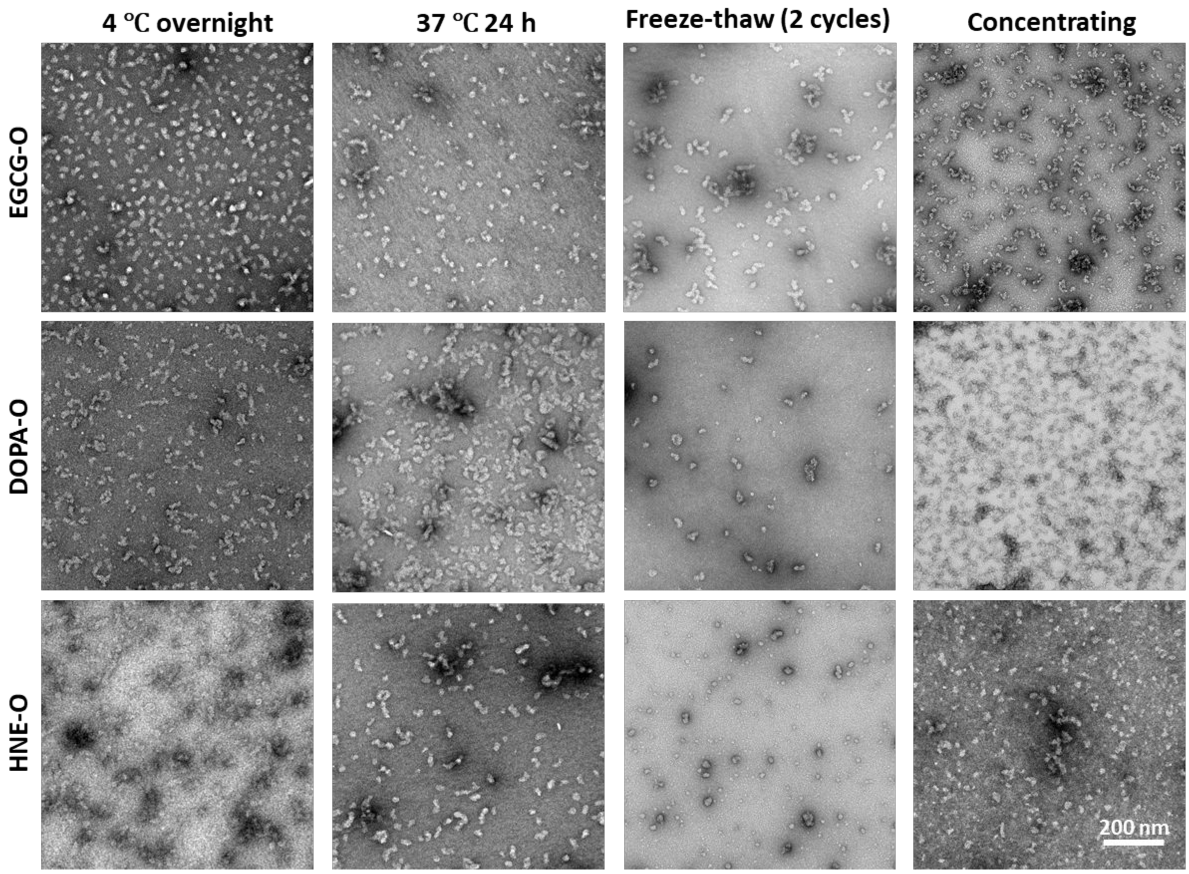

Figure S4. In vitro stability studies of chemically induced oligomers at 4 °C, 37 °C, freeze–thaw cycles and concentration using Amicon tubes. Scale bar: 200 nm.

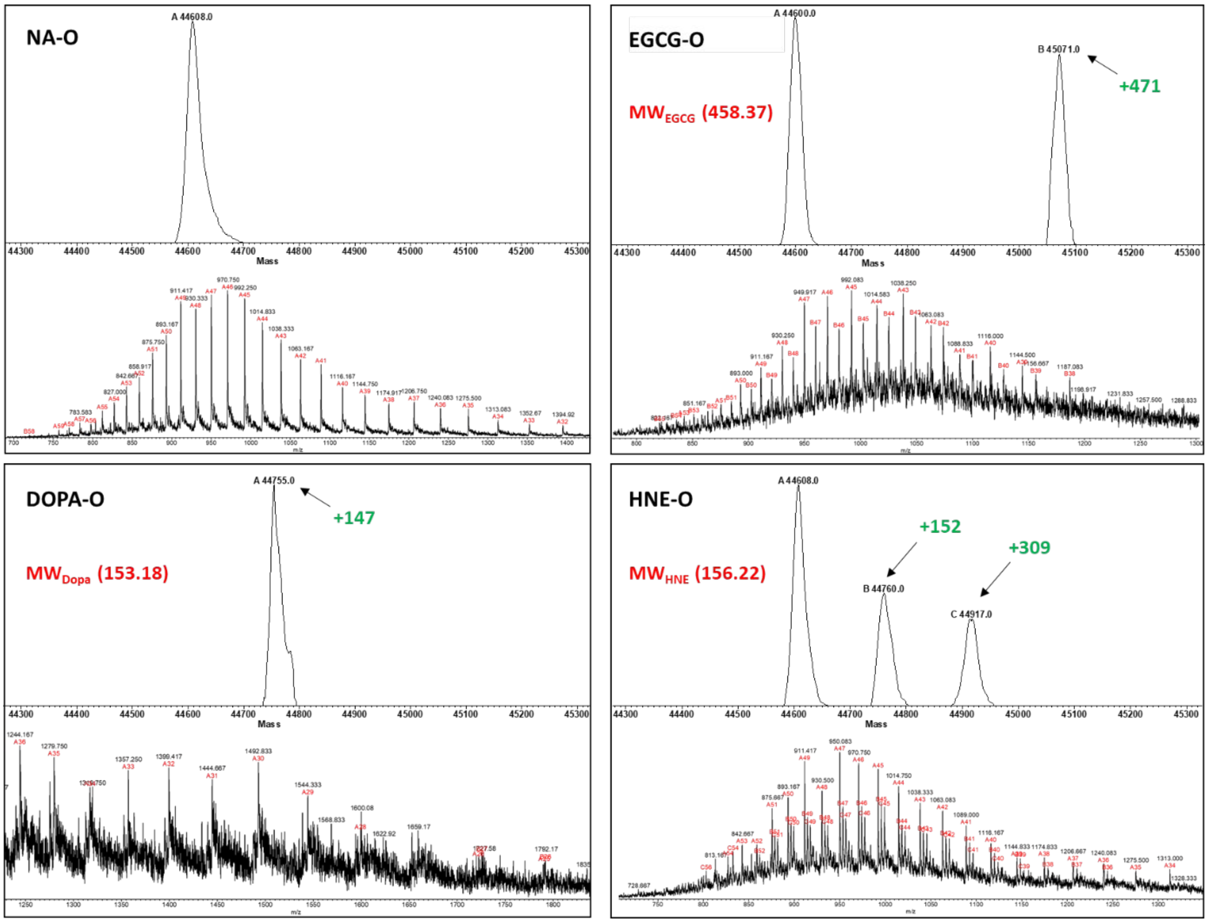

Figure S5. ESI-MS analysis of native and chemically induced TDP-43 oligomers.

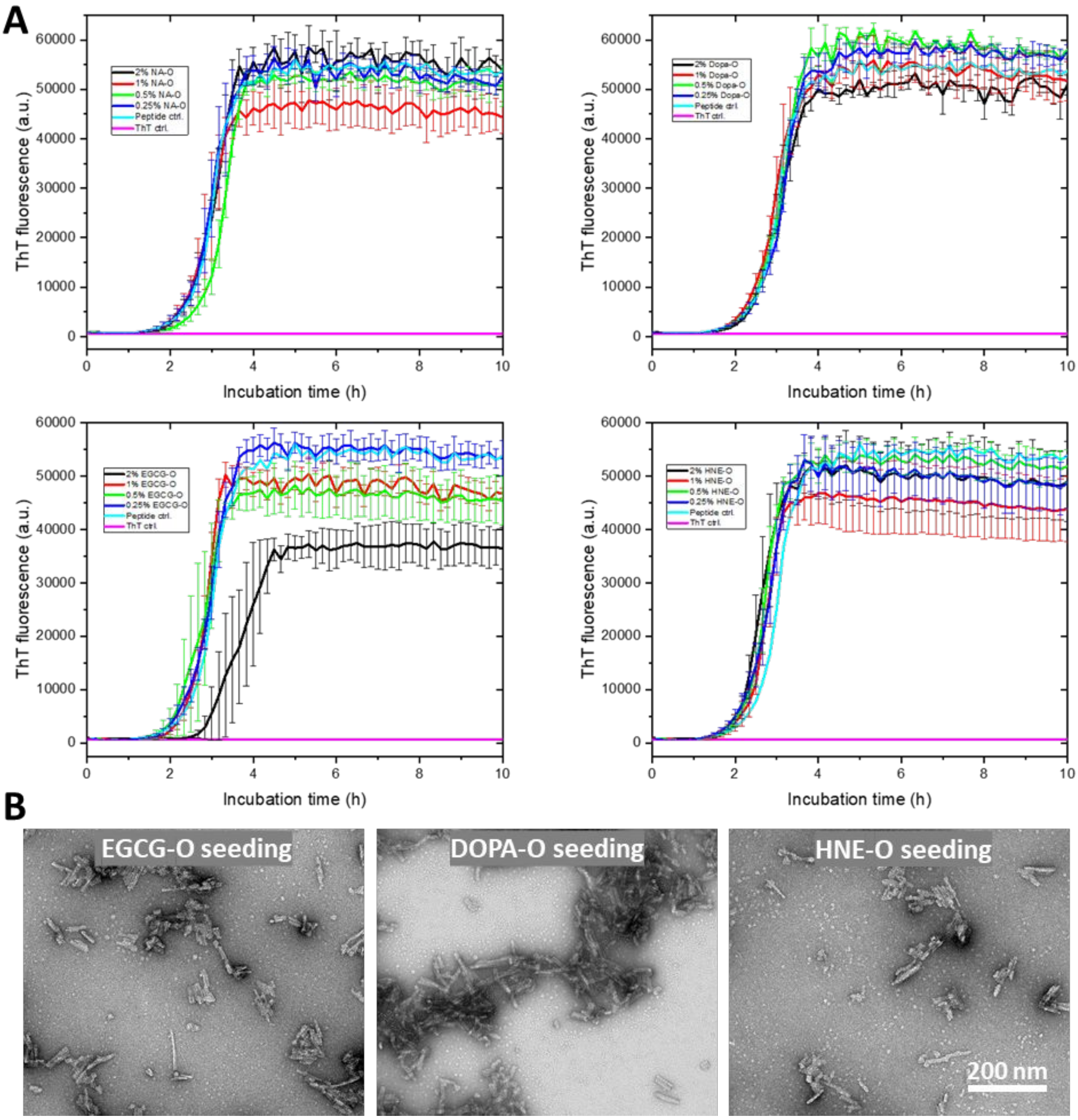

Figure S6. ThT kinetics-based seeding effect of TDP-43 oligomers on its core peptide 279-360 aggregation. (A) ThT kinetics using relatively low concentrations of TDP-43 oligomer seeds. (B) TEM analysis of the resulting samples after TDP-43 oligomer seeded aggregation of the core peptide. Scale bar: 200 nm.

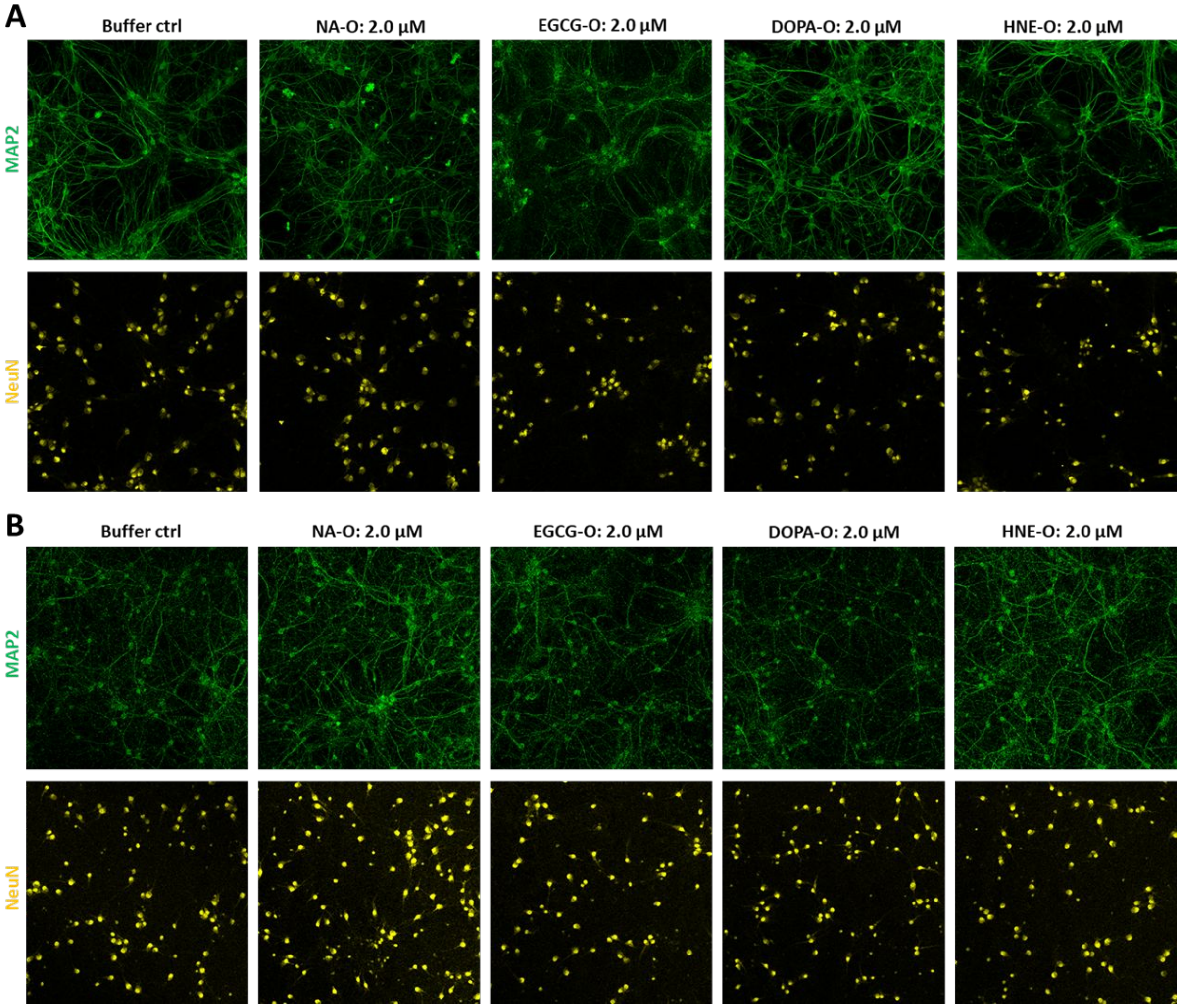

Figure S7. Cytotoxicity studies of different TDP-43 oligomers. Imaging of hippocampal neurons (A) and cortical neurons (B) after LDH assays. Immunocytochemistry of primary neurons treated with 2 μM TDP-43 oligomers. The samples were subjected to microtubule-associated protein-2 (MAP-2) immunostaining (green), neuronal nuclei (NeuN) immunostaining (yellow), and DAPI staining (blue, not shown).
